# Population structure of four Thai indigenous chicken breeds

**DOI:** 10.1186/1471-2156-15-40

**Published:** 2014-03-27

**Authors:** Supamit Mekchay, Pantaporn Supakankul, Anunchai Assawamakin, Alisa Wilantho, Wanwisa Chareanchim, Sissades Tongsima

**Affiliations:** 1 Department of Animal and Aquatic Sciences, Faculty of Agriculture, Chiang Mai University, Chiang Mai 50200, Thailand; 2 Center of Excellence on Agricultural Biotechnology: (AG-BIO/PERDO-CHE), Bangkok 10900, Thailand; 3 School of Agriculture and Natural Resources, University of Phayao, Phayao 56000, Thailand; 4 Department of Pharmacology, Faculty of Pharmacy, Mahidol University, Bangkok 10400, Thailand; 5 Biostatistics and Informatics Laboratory, Genome Institute, National Center for Genetic Engineering and Biotechnology (BIOTEC), Khlong Nueng, Khlong Luang, Pathum Thani 12120, Thailand

**Keywords:** Thai indigenous chicken, Population structure, Genetic variation, SNP, AFLP

## Abstract

**Background:**

In recent years, Thai indigenous chickens have increasingly been bred as an alternative in Thailand poultry market. Due to their popularity, there is a clear need to improve the underlying quality and productivity of these chickens. Studying chicken genetic variation can improve the chicken meat quality as well as conserving rare chicken species. To begin with, a minimal set of molecular markers that can characterize the Thai indigenous chicken breeds is required.

**Results:**

Using AFLP-PCR, 30 single nucleotide polymorphisms (SNPs) from Thai indigenous chickens were obtained by DNA sequencing. From these SNPs, we genotyped 465 chickens from 7 chicken breeds, comprising four Thai indigenous chicken breeds- Pradhuhangdum (PD), Luenghangkhao (LK), Dang (DA) and Chee (CH), one wild chicken - the red jungle fowls (RJF), and two commercial chicken breeds - the brown egg layer (BL) and commercial broiler (CB). The chicken genotypes reveal unique genetic structures of the four Thai indigenous chicken breeds. The average expected heterozygosities of PD= 0.341, LK= 0.357, DA=0.349 and CH= 0.373, while the references RJF= 0.327, CB=0.324 and BL= 0.285. The F_ST_ values among Thai indigenous chicken breeds vary from 0.051 to 0.096. The F_ST_ values between the pairs of Thai indigenous chickens and RJF vary from 0.083 to 0.105 and the F_ST_ values between the Thai indigenous chickens and the two commercial chicken breeds vary from 0.116 to 0.221. A neighbour-joining tree of all individual chickens showed that the Thai indigenous chickens were clustered into four groups which were closely related to the wild RJF but far from the commercial breeds. Such commercial breeds were split into two closely groups. Using genetic admixture analysis, we observed that the Thai indigenous chicken breeds are likely to share common ancestors with the RJF, while both commercial chicken breeds share the same admixture pattern.

**Conclusion:**

These results indicated that the Thai indigenous chicken breeds may descend from the same ancestors. These indigenous chicken breeds were more closely related to red jungle fowls than those of the commercial breeds. These findings showed that the proposed SNP panel can effectively be used to characterize the four Thai indigenous chickens.

## Background

The characterization of chicken genetic variation is very important for the investigation of genetic diversity within and between chicken populations as well as better our understanding on chicken evolution. Informative molecular markers, such as microsatellites and single nucleotide polymorphisms (SNPs), can greatly facilitate the assessment of the genetic diversity of the wild and domestic chickens. Several lines of evidence revealed that the ancestors of modern chickens arose out of South and Southeast Asia. Chicken mitochondrial DNA sequence indicated that the domestic chicken breeds have descent from the red jungle fowls (*Gallus gallus gallus*) in Southeast Asia [1,2]. Hillel *et al*. [3] showed by using microsatellite data that the red jungle fowls were the main progenitor and the major contributor of the domestic chicken gene pool. Furthermore, the domestic chickens have multiple maternal origins that occurred in South and Southeast Asia in area ranging from India, South China, Myanmar, Vietnam and Thailand [4].

Thai red jungle fowls (RJF) have been designated as an endangered species. They can only be found in national parks and forested mountains [5]. Several investigations were conducted on Thai red jungle fowls in which the DNA of these birds was sequenced and used as the reference for the genetic analysis in chickens [3,6-8]. Thailand has a rich genetic diversity of indigenous chickens. Four Thai indigenous chickens, namely Pradhuhangdum (PD), Luenghangkhao (LK), Dang (DA) and Chee (CH), represent Thai indigenous chickens that have been promoted as a chicken genetic resource for the purpose of conservation breeding and sustainable utilization of the chickens. Thai indigenous chicken breeds are becoming an increasingly important food source to those who live in rural areas in Thailand. Due to their uniqueness in meat quality, these chickens have become very popular among general consumers increasing their market prices to two or three times higher than the commercial broiler chickens [9]. The meat quality of these indigenous chickens has a unique taste with favorable toughness and hence, offering a low cholesterol and fat meat product [10]. Although there were several attempts to characterize these chickens using their physical properties and the successive proteomic profiling of the meat [9,11-13], only few research efforts have undertaken a genetic characterization of these indigenous chickens [4,14-17]. Up to the present, the genetic structure of Thai indigenous chickens remains unclear. Studying their population structures can better our understanding about their genetic makeups. A panel of genetic markers specific for identifying both indigenous and domestic chicken breeds can be identified, thus facilitating their conservation and breeding programs.

Microsatellite markers of chickens have previously been reported [5,6,14,15,18-20]. However, the limited number of these microsatellites may not capture the underlying genetic differences among closely related breeds. Furthermore, SNPs have obvious advantages in terms of cost effectiveness, ease of acquisition, higher coverage and they can provide a better estimate of genetic variation, for example, SNP genotyping was used in recent studies of genetic diversity of chicken breeds [8,21]. With the advent of parallel genotyping SNP arrays, SNP microarrays for chicken were developed [22,23]. However, their availability and accessibility are still limited to a few research centers in which several molecular techniques are required to genotype chicken SNPs, such as PCR-RFLP, Sequenom Mass Array and DigiTag2. These techniques can be used to successfully characterize several chicken populations [24-26]. Amplified fragment length polymorphism (AFLP) is an alternative technique to generate a large number of loci without prior knowledge of genome sequencing information [27,28]. The AFLP method has been used to characterize the genetic variation of several chicken breeds [29,30]. Moreover, their AFLP markers can be converted to simple codominant locus-specific (e.g. SNP) markers.

This study aims to develop a novel SNP panel derived from the AFLP technique and uses different genetic profiles derived from this SNP panel to characterize Thai the aforementioned indigenous chickens as well as the other reference breeds including red jungle fowls, commercial broiler and brown egg layer chicken breeds.

## Results and discussion

### SNP marker development

A total of 72 polymorphic AFLP bands were detected among the four Thai indigenous chicken breeds. These polymorphic bands were from 30 codominant SNP markers. These SNPs were used in the chicken population genetic analysis. The average observed and expected heterozygosities of the 30 SNP markers for the seven chicken breeds were 0.345 (0.116 to 0.638) and 0.353 (0.116 to 0.491), respectively (Table 1). The F-statistics of each locus is shown in Table 1. The average inbreeding coefficient within population (F_IS_), fixation index of each population (F_ST_) and heterozygotes across population (F_IT_) were 0.033, 0.096 and 0.126, respectively.

**Table 1:** **Observed (H**_**o**_**) and expected (H**_**e**_**) heterozygosity and F-statistics (F**_**IS**_**, F**_**ST **_**and F**_**IT**_**) for all loci across seven chicken breeds**

**Markers**	**H**_**o**_	**H**_**e**_	**F**_**IS**_	**F**_**ST**_	**F**_**IT**_
AFLP01	0.139	0.338	−0.139	0.210	0.100
AFLP02	0.116	0.190	−0.493	0.159	−0.256
AFLP03	0.433	0.435	−0.448	0.126	−0.266
AFLP04	0.271	0.302	0.529	0.068	0.561
AFLP05	0.373	0.375	0.386	0.022	0.399
AFLP06	0.485	0.469	0.026	0.034	0.059
AFLP07	0.465	0.431	0.058	0.170	0.218
AFLP08	0.460	0.431	0.032	0.199	0.225
AFLP09	0.246	0.216	−0.026	0.079	0.056
AFLP10	0.411	0.308	−0.073	0.054	−0.015
AFLP11	0.463	0.458	−0.056	0.061	0.008
AFLP12	0.118	0.242	−0.147	0.056	−0.082
AFLP13	0.400	0.403	−0.346	0.087	−0.229
AFLP14	0.235	0.297	0.012	0.154	0.164
AFLP15	0.246	0.291	0.518	0.037	0.536
AFLP16	0.374	0.387	0.037	0.043	0.079
AFLP17	0.258	0.300	0.239	0.050	0.277
AFLP18	0.288	0.358	0.185	0.040	0.217
AFLP19	0.195	0.491	0.073	0.171	0.232
AFLP20	0.351	0.483	0.029	0.068	0.095
AFLP21	0.300	0.291	0.194	0.129	0.298
AFLP22	0.587	0.461	0.580	0.064	0.607
AFLP23	0.497	0.482	0.300	0.057	0.340
AFLP24	0.134	0.154	−0.006	0.008	0.002
AFLP25	0.125	0.116	−0.225	0.092	−0.113
AFLP26	0.323	0.269	−0.008	0.011	0.003
AFLP27	0.301	0.443	0.068	0.143	0.201
AFLP28	0.611	0.384	−0.087	0.076	−0.005
AFLP29	0.494	0.394	−0.210	0.033	−0.170
AFLP30	0.638	0.387	0.302	0.172	0.422
Average	0.345	0.353	0.033	0.096	0.126

These results indicate that the proposed SNPs can effectively be used to classify the seven chicken breeds. The average observed and expected heterozygosities and the F_IS_ values of the seven chicken populations are shown in Table 2. From the heterozygosity perspective, the average of the heterozygosity values taken from all 30 SNPs (PD = 0.341, LK = 0.358, DA = 0.350, CH = 0.373, RJF = 0.327, BL = 0.324 and CB = 0.285) which is consistent with previous reports [5,16] that indicated the average expected heterozygosities of the local chicken populations and RJF in Southeast Asia regions were 0.309 to 0.395. In our study, however, the average expected heterozygosities of commercial broiler and brown egg layer chickens were lower than the chickens in [5,16], but they were higher than the broiler and layer chickens in the investigation of Shimogiri *et al.*[25]. Lower average expected heterozygosities were also observed in Indonesian indigenous chickens [24].

**Table 2:** **Average observed (H**_**o**_**) and expected heterozygosity (H**_**e**_**) and F**_**IS **_**values of Thai indigenous chickens, red jungle fowls and commercial chicken breeds**

Chicken breeds^*^	**No. of samples**	**H**_**o**_	**H**_**e**_	**F**_**IS**_
PD	100	0.340	0.341	0.002
LK	100	0.343	0.358	0.042
DA	100	0.294	0.350	0.161
CH	100	0.402	0.373	−0.076
RJF	20	0.328	0.327	−0.004
BL	25	0.312	0.324	−0.038
CB	20	0.314	0.285	−0.103

^*^ PD = Pradhuhangdum; LK = Luenghangkhao; DA = Dang; CH = Chee; RJF = red jungle fowl; BL = brown egg layer; CB = commercial broiler.

### Genetic diversity of chickens

Table 3 shows the pairwise F_ST_ values of the seven chicken populations. Among Thai indigenous chicken breeds, the PD breed was most closely related to the CH and LK breeds (F_ST_ = 0.051 and 0.059). The DA chicken was different from other the Thai indigenous chicken breeds (0.072 ≤ F_ST_ ≤ 0.096). Moreover, the CH and PD breeds were closely related to the red jungle fowls with F_ST_ = 0.083 and 0.091, respectively. The two commercial chicken breeds, CB and BL, were closely related (F_ST_ = 0.075) whereas; these two chicken breeds were separated from the Thai indigenous chickens and the red jungle fowls (0.116 ≤ F_ST_ ≤ 0.221). The permutation test on pairwise F_ST_ was used at 480 permutations with the Bonferroni correction. All F_ST_ values presented in Table 3 passed the cutoff with *P*-value <0.002 at 5% confidence interval. These findings indicated that Thai indigenous chicken breeds were more closely related to red jungle fowls than those from commercial broiler and layer chickens.

**Table 3:** **Pairwise fixation index (F**_**ST**_**)**^**† **^**among Thai indigenous chickens, red jungle fowls and the two commercial chicken breeds**

Chicken breeds^*^	**PD**	**LK**	**DA**	**CH**	**RJF**	**BL**	**CB**
PD	0.000						
LK	0.059	0.000					
DA	0.096	0.072	0.000				
CH	0.051	0.070	0.093	0.000			
RJF	0.091	0.105	0.147	0.083	0.000		
BL	0.134	0.167	0.142	0.125	0.148	0.000	
CB	0.123	0.189	0.221	0.116	0.209	0.075	0.000

^†^All F_ST_ values in this table were significant at *P*-value < 0.002 after the permutation test with Bonferroni correction. ^*^ PD = Pradhuhangdum; LK = Luenghangkhao; DA = Dang; CH = Chee; RJF = red jungle fowl; BL = brown egg layer; CB = commercial broiler.

### Phylogenetic tree analysis

**Figure 1:**
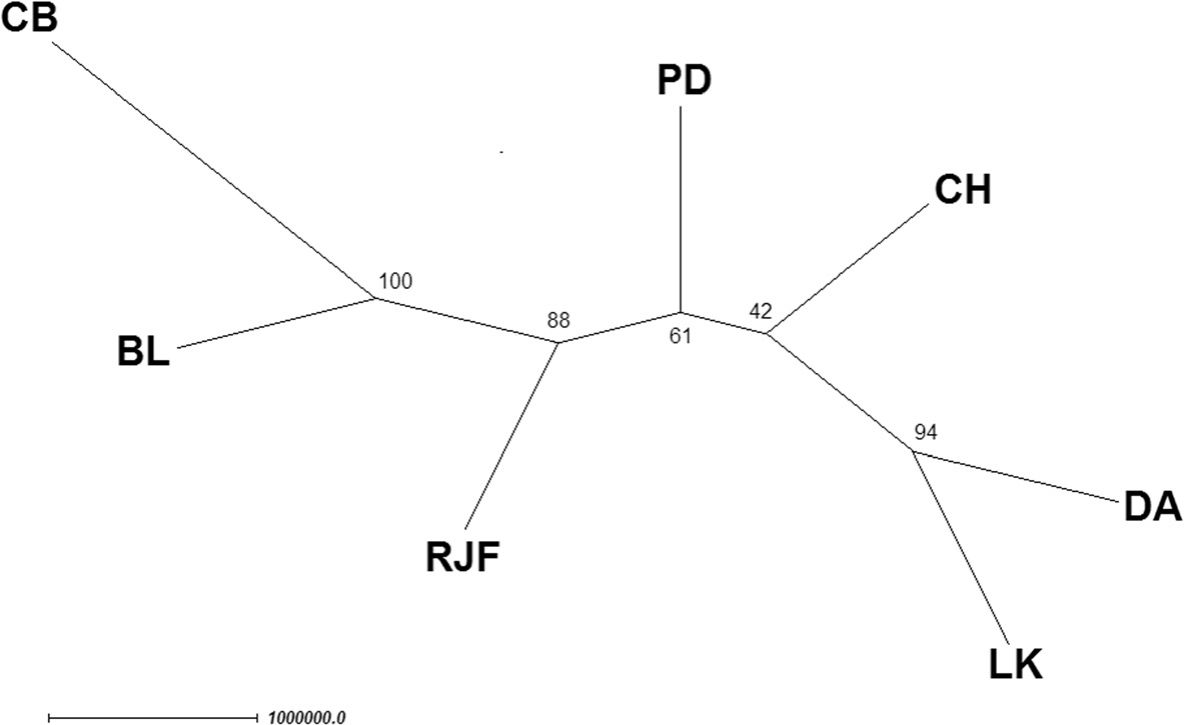
**The bootstrap NJ tree for the four Thai indigenous chicken breeds, red jungle fowls and two commercial chicken breeds.** The nodes in this phylogram represent the seven breeds, namely PD = Pradhuhangdum, LK = Luenghangkhao, DA = Dang, CH = Chee, RJF = red jungle fowl, BL = brown egg layer chicken, and CB = commercial broiler chicken breeds. The number of each branch represents the percentage of the bootstrap tree (1,000,000 bootstrapping). The branch length reflects the genetic distance between clades.

**Figure 2:**
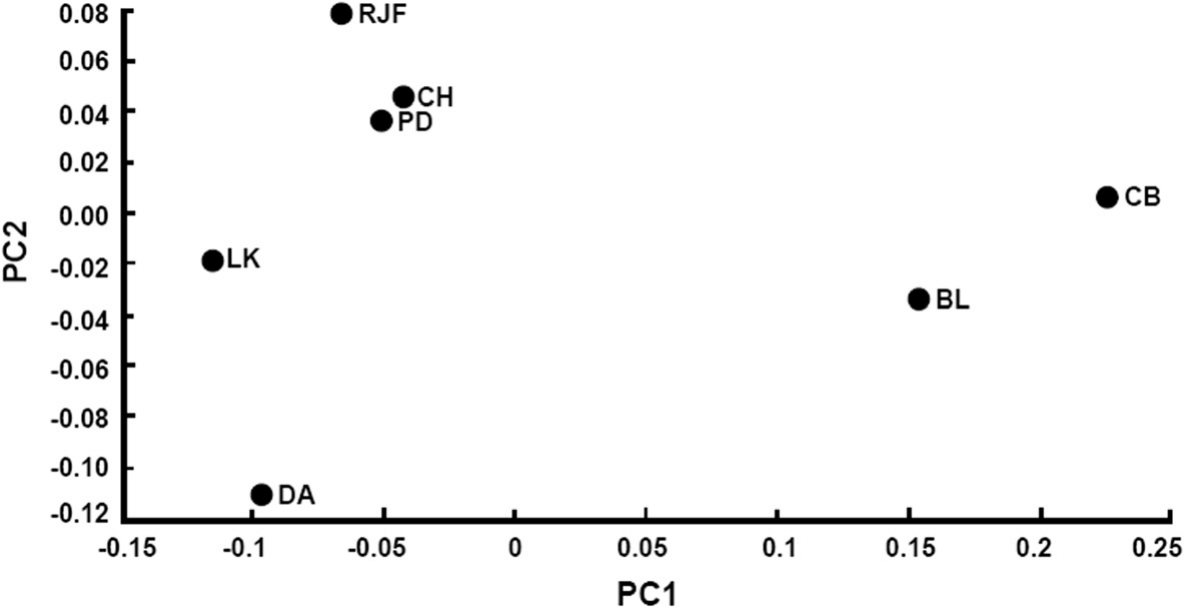
**Principal component analysis (PCA) between PC1 and PC2 of four Thai indigenous chicken breeds, red jungle fowls and two commercial chicken breeds.** The round black dots represent the seven breeds, namely PD = Pradhuhangdum, LK = Luenghangkhao, DA = Dang, CH = Chee, RJF = red jungle fowl, BL = brown egg layer chicken, and CB = commercial broiler chicken breeds.

A phylogenetic tree of the four Thai indigenous chickens, red jungle fowls and two commercial chicken breeds was constructed based on the Neighbour-Joining (NJ) algorithm (Figure 1). The bootstrapping values of this NJ tree vary from 47 to 100%. The resulting NJ tree bootstrapping range and the NJ tree topology are also consistent with the prevoius observations on domestic chicken breeds from Asia, Africa, South America, Finland and Spain as well as the commercial breeds (White Leghorn, Rhode Island Red and broiler) [18,19,31]. The Thai indigenous chickens were grouped together in the phylogram along with the red jungle fowls. The commercial chickens were separated into two groups (commercial broiler and brown egg layer chickens). These commercial chickens were placed to the branches whose distances are far from the Thai indigenous chickens and red jungle fowls. This NJ tree topology and branch lengths are also consistent with the PCA plot (Figure 2). The PCA plot showed that the PD and CH chicken breeds were most closely related with the red jungle fowls, whereas the commercial broiler and brown egg layer chickens were greatly separated from the four Thai indigenous chicken breeds and the red jungle fowls.

### Genetic structure of chicken

**Figure 3:**
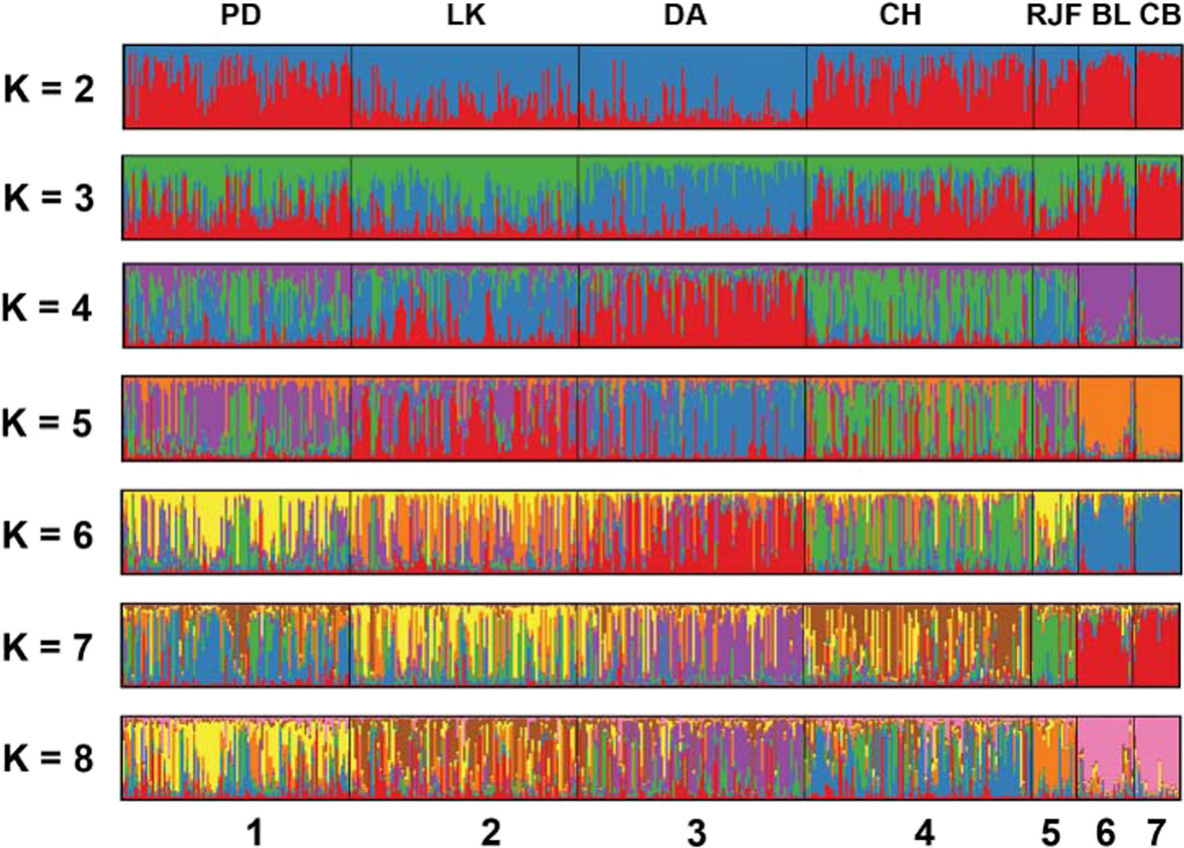
**Admixture plots of four Thai indigenous chicken breeds, red jungle fowls (RJF), brown egg layer (BL) and commercial broiler (CB) chickens with varying number of inferred ancestors K = 2 to K = 8.** Each vertical line represents the mixing of K proportions/colors (admixture) for a chicken. The order of chickens in each breed is arbitrary but chickens from the same breed are grouped together, where the numbers 1 to 7 represent different breeds, namely PD = Pradhuhangdum, LK = Luenghangkhao, DA = Dang, CH = Chee, RJF = red jungle fowl, BL = brown egg layer chicken, and CB = commercial broiler chicken breeds, respectively.

The population structure of the seven chicken breed populations was analyzed using STRUCTURE. Figure 3 shows the admixture plot of all individuals revealing the admixture patterns of these chicken breeds ranging from K = 2 to 8. At K = 2, the admixture plot of all chicken breeds reveals two distinct population patterns. Particularly, the combined DA and LK admixture plot represents a different pattern from those of PD, CH, RJF, BL and CB chicken breeds. At K = 3, DA exhibited different pattern observed in the LK group, whereas PD and CH revealed the admixture pattern similar to that of RJF, CB and BL chicken breeds. At K = 4, DA, LK and CH were clearly separated from the other chicken breeds, while PD’s pattern was inseparable with that of RJF. Both commercial breeds CB and BL appeared to be similar in terms of admixture pattern and this pattern was distinct from the other breeds. At K = 5 and K = 6, the admixture patterns revealed similar patterns to that of K = 4. At K = 7-8, all four Thai indigenous chickens appeared to have distinguishable admixture patterns. In particular, PD was clearly separated from the RJF chicken and two commercial are clearly separated from the other Thai indigenous chickens.

From eye inspection, the admixture plot at K = 7 could best describe the chicken structure in which the four Thai indigenous chickens were represented by four distinct admixture patterns (four predominating colors). These four patterns are clearly separated from the RJF and the two commercial breeds. This selected result is consistent with the estimated optimal K using maximum likelihood L(K) = −14597.83 at K = 7 and ΔK (at K = 7) was 3.42. These results indicate that Thai indigenous chicken breeds may descend from the same ancestor(s) and possibly the PD and CH are related to the red jungle fowls. Such genetic affiliation could result from intensive breeding selection for improving either meat or egg production in the commercial breeds. On the contrary, the indigenous breeds and the wild chicken RJF have not been under the aforementioned selection pressure [5]. Furthermore, the previous research [6,32] reported a potential gene flow from the jungle fowls to local Asian chicken breeds. The results in this study are consistent with the previous study which found that the domestic chickens were grouped with the red jungle fowls subspecies as well as the brown egg layer and broiler were clustered together in one branch based on 51,076 SNP markers information [8]. Furthermore, the works by [5,16] reported similarly that there exists stronger SNP association between Thai indigenous breeds and the red jungle fowls than the association with the commercial breeds. However, the reports by [14,15] based on microsatellite data showed a contradictory results that the commercial broiler breed was closer to the red jungle fowls. The contradiction may stem from the false positive result due to much less number of polymorphic markers as well as the population size and statistical analysis approach.

Although microsatellite markers have been widely used to analyze genetic diversity and structure of chicken breeds [3,17,19,33], microsatellite analysis can be affected by missing alleles and high variable mutation patterns [26]. With the advent of better genotyping technology, more than 70 SNPs can be used to investigate the genetic structure of chicken populations [24,25]. A more recent work by [8] used SNParray (58K markers) to cluster the genomes of commercial and non-commercial chicken breeds. It has been reported that the numbers of SNP markers are important for clustering analysis and the 22 most polymorphic SNP markers are sufficient to cluster the genetic structure of chickens with 95% accuracy of affiliation [26].

These findings highlight the importance of the selected SNP markers derived from AFLP that they could be used to characterize Thai indigenous chicken. Such information can be used to better our understanding about the genetics of these Thai indigenous chicken breeds as well as their relationships with the RJF and two commercial chicken breeds.

## Conclusions

The proposed SNP panel can be used to characterize four Thai indigenous breeds from the red jungle fowls and two commercial chicken breeds. Moreover, these indigenous breeds were more closely related to the red jungle fowls than the commercial broiler and brown egg layer chickens. Furthermore, the commercial broiler chickens revealed an admixture pattern similar to the commercial brown egg layer chickens. Such results suggest that the proposed 30 SNPs are very effective for characterizing Thai indigenous chickens. Finally, the genetic structure analysis indicates that the four Thai indigenous chicken breeds may descend from the same chicken ancestor(s).

## Methods

### DNA samples

Blood samples were taken from a total of 465 individual chickens from seven different breeds. Four Thai indigenous chicken breeds, consisting of Pradhuhangdum (PD, n =100), Luenghangkhao (LK, n =100) Dang (DA, n = 100) and Chee (CH, n = 100) were obtained from the Chiang Mai Livestock Breeding and Research Center, Chiang Mai Province, the Kabinburi Livestock Breeding and Research Center, Prachinburi province, the Surattani Livestock Breeding and Research Center, Surattani province and the Thapra Livestock Breeding and Research Center, Khon Kaen province, respectively. A red jungle fowl population (RJF, n = 20) was obtained from the Chiang Mai Zoo, Chiang Mai province. A brown egg layer chicken population (namely Isa Brown, BL, n = 25) and a commercial broiler chicken population (namely Arbor Acres, CB, n = 20) were obtained from a local company. DNA was extracted using a spin column-based PureLink® Genomic DNA Mini Kit (Invitrogen, CA). Forty DNA samples of Thai indigenous chicken breeds (10 birds of each breed) were arbitrarily recruited to locate distinguishable polymorphic loci to distinguish these chickens using AFLP. Then we performed targeted sequencing on these loci to identify novel SNPs that could be used to distinguish the selected breeds. The study was approved by the animal ethics committee of Faculty of Agriculture, Chiang Mai University.

### AFLP analysis

AFLP was carried out as described by Vos *et al*. [34] and Wimmers *et al*. [27]. The sequence of the adapter and primers were used as described in the previous study [35]. Genomic DNA (250 ng) was digested with *Taq*I (FastDigest^®^, Fermentas) at 65°C for 5 minutes and *Eco*RI (FastDigest^®^, Fermentas) at 37°C for 5 minutes. Adapters were ligated to the restriction fragments by a ligation reaction containing 1 U of T4 DNA ligase (Fermentas), 10 pmol of double-stranded *Eco*RI adapter and 100 pmol of double-stranded *Taq*I adapter. The reactions were incubated at 20°C for 3 hours and then at 4°C overnight [30]. The amplification condition was performed with two rounds of preamplification and selective amplification. The preamplification was carried out with an *Eco*RI-N primer (E + A) and the *Taq*I-N primer (T + C). The selective amplification was performed with 64 primer combinations (*Eco*RI-ANN and *Taq*I-CNN). The reaction was added with loading dye (98% formamide, 10 mM EDTA, 0.025% xylenecyanol and 0.025% bromophenol blue) and denatured at 95°C for 5 minutes then immediately cooled on ice. The AFLP products were separated with 6% denaturing polyacrylamide gel electrophoresis at constant power (50 W) for 3 hours. The AFLP fingerprints were visualized by silver staining.

### Cloning and sequencing of AFLP fragments

The AFLP bands of interest were excised from dried gel, incubated in 30 μl 1 x PCR buffer at 4°C overnight, and boiled at 95°C for 10 minutes. The DNA fragments were then reamplified using the same PCR conditions as for the selective amplification of the PCR reactions. The reamplified DNA fragments were purified with the QIAquick PCR purification kit (Qiagen, Germany) and cloned into a pGEM-T easy vector (Promega, USA). Recombinated clones were sequenced with the CEQ 8000 Genetic Analysis System (Beckman-Coulter). The nucleotide sequences were compared with the Ensembl database (http://asia.ensembl.org/Multi/blastview).

### Conversion AFLP to SNP markers

The polymorphic bands of the AFLP markers were sequenced. These nucleotide sequences were compared with the chicken genome database using BLAST software [36]. Single tagged sequence (STS) markers obtained from different chicken breeds were sequenced to discover the polymorphic sites within the AFLP fragments. In order to identify simple codominant SNP markers, the NEBcutter software (http://tools.neb.com/NEBcutter2) was used to locate the specific restriction enzymes for each SNP markers.

### SNP genotyping

The PCR products were performed in 20 μl containing 50 ng of genomic DNA, 0.4 μM of forward and reverse primers, 50 μM of dNTPs, 1.5 mM MgCl_2_ 0.25 U *Taq* polymerase (Fermentas) and 1 x PCR buffer. The PCR condition was 94°C for 3 minutes as an initial denaturation and 35 cycles of 94°C for 30 sec, 55–60°C for 30 sec, 72°C for 60 sec and final extension at 72°C for 5 minutes. The PCR products (5 μl) were digested with restriction enzymes. The restriction fragments were separated using 6% polyacrylamide gels and visualized with silver staining. A list of the primers used for SNP marker amplification and the restriction enzymes are shown in Table 4.

**Table 4:** Location of AFLP sequences, list of primers and restriction enzymes for genotyping of SNP markers

**Markers**	**Chromosome**	**Forward primer**	**Reverse primer**	**Annealing Tem. (°C)**	**Enzyme**
AFLP01	2	5*'*-tgtatttccaacctactctac-3*'*	5*'*-gctgtacgtaaggctgcag-3*'*	58	*Taq*I
AFLP02	3	5*'*-gcacctggaagaatagatag-3*'*	5*'*-gtctttcctggtattcttatg-3*'*	56	*Eco*RI
AFLP03	4	5*'*-caggaacagcatagaattaaag-3*'*	5*'*-caccagtgaccaaggcaag-3*'*	58	*Taq*I
AFLP04	15	5*'*-agctgctctcttcagtcag-3*'*	5*'*-agatttcaaagtgtcatgtgc-3*'*	58	*Taq*I
AFLP05	4	5*'*-tgcaatgcttatctacctgg-3*'*	5*'*-cgtctatctggaaattgctg-3*'*	58	*Taq*I
AFLP06	11	5*'*-gagaacctgctggtggtg-3*'*	5*'*-tccatgtggcggacgatg-3*'*	60	*Hsp*92II
AFLP07	4	5*'*-gtttcagcaaggcagagttc-3*'*	5*'*-gaccaaacaacgtcacgttc-3*'*	58	*Taq*I
AFLP08	6	5*'*-tttgtttcagcaaggcagag-3*'*	5*'*-gaccaaacaacgtcacgttc-3*'*	58	*Taq*I
AFLP09	1	5*'*-aaccatgcagccagagaatg-3*'*	5*'*-gcagccattcatacatggtc-3*'*	58	*Eco*RI
AFLP10	14	5*'*-gctctgatcaaagacattcc-3*'*	5*'*-gatcatcttcggctacagag-3*'*	58	*Alu*I
AFLP11	11	5*'*-gtgcagctggggttggg-3*'*	5*'*-ggaaccggtgcttgcattg-3*'*	58	*Bsu*RI
AFLP12	1	5*'*-tgaccatgctttctccatag-3*'*	5*'*-tttggaaatcaattttcagctc-3*'*	58	*Taq*I
AFLP13	1	5*'*-gcagctgcgtataaacacag-3*'*	5*'*-caggactgcagggataaatg-3*'*	58	*Taq*I
AFLP14	10	5*'*-gcttcagcaggcagatttc-3*'*	5*'*-ctttacgtggcccaccttc-3*'*	58	*Bsu*RI
AFLP15	2	5*'*-aaactcttttcccttggctg-3*'*	5*'*-gtgcaagacggactgtattg-3*'*	58	*Eco*RI
AFLP16	8	5*'*-actcgcaggagaacttcttag-3*'*	5*'*-gcagtcctgtgacttatttg-3*'*	58	*Eco*RI
AFLP17	1	5*'*-gaaatcgttgccaaaagttgc-3*'*	5*'*-gttcacgcagctcggatg-3*'*	58	*Msp*I
AFLP18	7	5*'*-ggtttgatttctgggatctc-3*'*	5*'*-gccttaggtaacattccttc-3*'*	58	*Eco*RI
AFLP19	2	5*'*-cctcctactgattctgtaatg-3*'*	5*'*-ttttctgctcatctgtactgg-3*'*	56	*Hin*6I
AFLP20	4	5*'*-aggatcacaaataaccaacga-3*'*	5*'*-gactacagtgagaagctctg-3*'*	60	*Mbo*I
AFLP21	10	5*'*-gtctgcacacctggtgtc-3*'*	5*'*-caggttcacacggagatc-3*'*	58	*Msp*I
AFLP22	1	5*'*-ggaggttcgtgagaagctg-3*'*	5*'*-tgtacaacagcagcaagcaaa-3*'*	60	*Hsp*92II
AFLP23	8	5*'*-ctttctccttctccccaagt-3*'*	5*'*-cttggatagggtctgcaga-3*'*	58	*Taq*I
AFLP24	1	5*'*-gcagtgcacctggattttag-3*'*	5*'*-attatcccttccctcagctg-3*'*	60	*Taq*I
AFLP25	17	5*'*-ctgctagcaggtaatgagat-3*'*	5*'*-tggcagaaagattccgtcaa-3*'*	55	*Taq*I
AFLP26	2	5*'*-tctctgctagtgtgtgtgga-3*'*	5*'*-caccaggactgaagaacaga-3*'*	60	*Eco*RI
AFLP27	3	5*'*-ctgagaatagccaggacaca-3*'*	5*'*-gtgttgggaatttaggaaac-3*'*	58	*Taq*I
AFLP28	11	5*'*-gtgagggcaaccagagcca-3*'*	5*'*-tgaagattcctgttcttgag-3*'*	58	*Hsp*92II
AFLP29	9	5*'*-ctgctgcggaccgaaatatc-3*'*	5*'*-gatagttccgaacagtttgc-3*'*	58	*Bsu*RI
AFLP30	13	5*'*-cagatgatcacagtaacctg-3*'*	5*'*-gtggaacttgtaagtacgc-3*'*	58	*Taq*I

### Statistical analysis

Both the observed and expected heterozygosity values were calculated according to Freeland [37]. The inbreeding coefficient (F_IS_), fixation index of each population (F_ST_) and heterozygotes across population (F_IT_) were estimated using the FSTAT 2.9.3 [38] and GENEPOP 4.0 programs [39]. A neighbour-joining (NJ) tree of the seven chicken populations was constructed using PHYLIP version 3.69 [40]. The robustness of each clade in the NJ tree was assessed using 1,000,000 bootstrapping. Principal component analysis (PCA) was performed to analyze the genetic distance matrix of the seven chicken breeds using Matlab (R2009b). The genetic structure of the seven chicken populations was analyzed using STRUCTURE 2.3.3 [41]. A total of 30 SNPs were analyzed using the admixture model with 100,000 burn-ins followed by 20,000 Markov chain Monte Carlo (MCMC) replicates for varying number of inferred ancestors, K = 2 to K = 8. CLUMPP version 1.1.2 was used to estimate population structure after several runs [42]. The admixture plots were rendered using the DISTRUCT program [43]. To predict the optimal number of inferred ancestors (K), the maximum likelihood L(K) and the rate of change of the likelihood function with respect to K (ΔK) were computed from 50 runs of STRUCTURE for each K [44] using Structure Harvester v.0.6.93 [45].

## Competing interests

The authors declare that they have no competing interests.

## Authors’ contributions

SM designed experiment and performed the research as well as data interpretation and wrote the manuscript. PS carried out AFLP assay. AA, AW, ST and WC analyzed data. ST designed the bioinformatics workflow, interpreted the results and edited manuscript. All the authors have read and approved the final manuscript.
